# Contribution of p62 to Phenotype Transition of Coronary Arterial Myocytes with Defective Autophagy

**DOI:** 10.1159/000457877

**Published:** 2017-02-03

**Authors:** Junxiang Bao, Guangbi Li, Xinxu Yuan, Erich Gulbins, Pinlan Li

**Affiliations:** a Department of Pharmacology and Toxicology, Virginia Commonwealth University, School of Medicine, Richmond, VA, USA,; b Department of Molecular Biology, University of Duisburg-Essen, Essen, Germany

**Keywords:** Autophagy, Vascular smooth muscle cell, Arteriosclerosis, Differentiation, CD38

## Abstract

**Background:**

Autophagy disorder contributes to dedifferentiation of arterial smooth muscle cells, but the mechanisms are poorly understood. Here, we sought to investigate the role of scaffolding adaptor p62/SQSTM1 (p62) in phenotype switching of mouse coronary arterial myocytes (CAMs) induced by CD38 gene deficiency or lysosomal dysfunction which blocks autophagic flux in the cells.

**Methods:**

Protein expression was measured by western blot analysis and immunofluorescent staining. Cell cycle and proliferation rate were analyzed by flow cytometry and MTS assay respectively. mRNA abundance was tested by qRT-PCR.

**Results:**

CD38 gene deficiency or bafilomycin A1 (baf), a selective lysosomal inhibitor treatment increased proliferation rate and vimentin expression in CAMs which was prevented by p62 gene silencing. Cell percentage in G_2_/M and G_0_/G_1_ phase was decreased and increased by CD38 deficiency or baf treatment, respectively which was accompanied by accrual of cyclin-dependent kinase 1 (CDK1) protein. Although free ubiquitin content was increased, the colocalization of it to CDK1 was markedly decreased in CD38^−/−^ or baf treated CAMs. Furthermore, the changes in both cell cycle and CDK1 ubiquitinylation could be restored by p62 gene silencing.

**Conclusion:**

The results suggest in CD38^−/−^ or baf treated CAMs, p62 accumulation promotes phenotype transition and proliferation by accelerating cell cycle progress through G_2_/M which might relate to the compromised ubiquitinylation and degradation of CDK1.

## Introduction

Autophagy is a critical cellular homeostatic process through which unnecessary or dysfunctional components of cells are degraded for recycling or removal. This process may be either protective or detrimental for atherosclerosis, depending upon the status of autophagy or stages of the disease [1]. Well controlled autophagy in the arterial wall may help clean up damaged components and recover cells from atherogenic impairment which might lead the vascular smooth muscle cells (VSMCs) to a differentiated, quiescent, and contractile phenotype [2]. On the contrary, defective autophagy promotes the proliferation and dedifferentiation of VSMCs contributing to the generation and deterioration of atherosclerosis [3, 4].

CD38 is a multifunctional enzyme responsible for the production and metabolism of cyclic ADP-ribose (cADPR) and nicotinic acid adenine dinucleotide phosphate (NAADP) in VSMCs, which regulates vascular functions through modulating Ca^2+^ concentration ([Ca^2+^]) within cells [5]. Our previous studies have shown that NAADP-mediated release of Ca^2+^ from lysosomes and consequent increase of cytosolic [Ca^2+^] accelerated autophagosome trafficking and fusion with lysosomes, a process determining autophagic flux in mouse coronary arterial myocytes (CAMs). We also detected that CD38 deficiency (CD38^−/−^) or lysosomal malfunction dampened autophagy inducing proliferation and dedifferentiation of CAMs upon atherogenic stimulation [6]. However, mechanism by which the compromised autophagy evokes proliferation and phenotype switching in CD38^−/−^ CAMs or that with lysosomal dysfunction is still unclear.

Scaffolding adaptor p62/SQSTM1 (p62), a stress inducible intracellular protein [7] acts as a cargo receptor or adaptor participating in autophagic degradation of ubiquitinylated substrates. The domain architecture of p62 contains a N-terminal *phox/*bem1p (PB1) domain, a C-terminal ubiquitin-associated (UBA) domain and a microtubule-associated protein light chain 3 (LC3)-interacting region (LIR) motif through which p62 interacts with autophagy-related protein 8 (ATG8) family and ubiquitinated proteins [8]. The p62 mediated selective autophagic clearance of proteins provides an important degradation mechanism, especially when organisms are under the influence of mutation, aging, or environmental stresses [9]. A defect in autophagy may result in an p62 accumulation associated with a host of detrimental effects including rapid tumor growth with adverse prognostic behavior [10], distant metastases [11], neuropathology [12], and neurodegenerative disorders [13]. In the present study, we sought to elucidate whether the p62 accumulation elicited the proliferation and phenotype change of CAMs following autophagy defect induced by CD38 gene deficiency or lysosomal disorder.

With *in vitro* and *in vivo* approaches, our data demonstrated that the proliferation and dedifferentiation of CAMs induced by CD38 gene deficiency or lysosomal dysfunction was reversed by p62 gene silencing. We also found the G_2_/M phase progress in cell cycle analysis was enhanced after autophagy was prevented which was accompanied by attenuated ubiquitinylation and degradation of cyclin-dependent kinase 1 (CDK1). The changes in cell cycle and CDK1 ubiquitinylation could also be normalized after p62 gene was suppressed. Our work may raise a novel signaling pathway linking autophagy disorder with proliferation or phenotype switching in VSMCs of coronary artery, especially under atherogenic stimuli.

## Material and Methods

### Animal procedure

Both CD38^−/−^ and CD38^+/+^ mice on C57BL/6 background were purchased from the Jackson Laboratory. CD38^+/+^ and CD38^−/−^ mice (8 weeks of age; male) were fed 10-week standard rodent chow or Western diet containing (g%): protein 20, carbohydrate 50, and fat 21 (Dytes, Bethlehem, PA, USA), as described previously [14]. Mice were sacrificed by cervical dislocation under isoflurane anesthesia. The hearts with the coronary artery were obtained to be frozen in liquid nitrogen for preparation of frozen section slides. All experimental protocols were reviewed and approved by the Animal Care Committee of the Virginia Commonwealth University. All animals were provided water ad libitum and housed in a temperaturecontrolled room.

### Primary cell culture of mouse CAMs

CAMs were isolated from CD38^+/+^ or CD38^−/−^ mice as previously described [15]. In brief, mice were deeply anesthetized with an intraperitoneal injection of pentobarbital sodium (25 mg/kg). The heart was excised with an intact aortic arch and immersed in a petri dish filled with ice-cold Krebs-Henseleit solution. A 25-gauge needle filled with Hanks’ buffered saline solution (HBSS) was inserted into the aortic lumen opening while the whole heart remained in the ice-cold buffer solution. The opening of the needle was inserted deep into the heart close to the aortic valve. The needle was tied in place with the needle tip as close to the base of the heart as possible. The infusion pump was started with a 20 ml syringe containing warm HBSS through an intravenous extension set at a rate of 0.1 ml/min for 15 min. HBSS was replaced with warm enzyme solution (1 mg/ml collagenase type I, 0.5 mg/ml soybean trypsin inhibitor, 3% bovine serum albumin (BSA), and 2% antibiotic), which was flushed through the heart at a rate of 0.1 ml/min. Perfusion fluid was collected at 30-, 60-, and 90-min intervals. At 90 min, the heart was cut with scissors, and the apex was opened to flush out the cells that collected inside the ventricle. The fluid was centrifuged at 1000 rpm for 10 min, and then the cell-rich pellets were mixed with media. Next, the cells were plated on 2% gelatin-coated six-well plates and incubated in 5% CO_2_ at 37 °C. Advanced Dulbecco’s modified Eagle’s medium (DMEM) with 10% fetal bovine serum, 10% mouse serum, and 2% antibiotics was used for isolated smooth muscle cells. The identification of CAMs was based on positive staining by anti-α-actin antibody and the SMC morphology. The medium was replaced 3 days after cell isolation and then once or twice each week until the cells grew to confluence. All studies were performed with cells of passage of 3–5.

### Western blot analysis

Western blot analysis was performed as described previously [6]. In brief, proteins from the CAMs or dissected coronary arteries were separated by 12% sodium dodecyl sulfate-polyacrylamide gel electrophoresis (SDS-PAGE). The proteins of these samples were then electrophoretically transferred onto a PVDF membrane (Millipore, USA). The membrane was blocked with 5% nonfat milk and then probed with primary antibodies overnight at 4°C, followed by incubation with horseradish peroxidase-labeled IgG (1:5000). Primary antibodies used were mouse anti-vimentin (1:1000, Abcam), rabbit anti-α-smooth muscle actin (SMA, 1:1000, Abcam), rabbit anti-calponin (1:1000, Abcam), rabbit anti-p62 (1:200, Abcam), mouse anti-ubiquitin (1:1000, Cell Signaling), rabbit anti-CDK1 (1:1000, Cell Signaling) or goat anti-β-actin (1:2000, Santa Cruz). The immuno-reactive bands were detected by chemiluminescence methods and visualized on Kodak Omat X-ray films. Densitometric analysis of the images obtained from X-ray films was performed using the Image J software (NIH).

### Immunofluorescent staining of CAMs

Cultured CAMs were grown on glass coverslips, stimulated or left unstimulated and fixed in 4% paraformaldehyde in phosphate-buffer saline (PFA/PBS) for 15 min. After permeabilization with 0.1% Triton X-100/PBS, the cells were rinsed with PBS and incubated overnight at 4°C with indicated primary antibodies: mouse anti-vimentin (1:200, Abcam), rabbit anti-α-SMA (1:200, Abcam), rabbit anti-calponin (1:200, Abcam), rabbit anti-p62 (1:200, Abcam), mouse anti-ubiquitin (1:200, Cell Signaling) and rabbit anti-CDK1 (1:200, Cell Signaling). The slides were extensively washed and incubated with Alexa-488- or Alexa-555-labeled secondary antibodies for 1 h at room temperature. The slides were mounted and subjected to examinations by using sequentially scanning on a laser scanning confocal microscope (Fluoview FV1000, Olympus, Japan), with photos being taken, and the co-localization analyzed by the Image Pro Plus 6.0 software (Media Cybernetics, Bethesda, MD, USA). The summarized co-localization efficiency data was expressed as Pearson correlation coefficient (PCC) as described previously [16].

### Cellular proliferation analysis

Cell proliferation was measured as we previously described [17]. The cultured CAMs were adjusted to density of 1×10^4^/mL in 100 μl of culture medium with or without treatment in a 96-well assay plate. After 48 h, 20 μL of CellTiter 96^®^ Aqueous One Solution Cell Proliferation Assay (MTS) (Promega) were added. Then, the plate was incubated at 37°C for 1h in a humidified, 5% CO_2_ atmosphere and the absorbance at 490 nm was recorded using a 96-well plate reader. Calibration curves showed the fluorescence reading to be proportional to the cell number. The proliferation rate was obtained by calculating the fold change in the cell number of each sample before and after 48-h incubation.

### Cell cycle analysis

The CAMs were synchronized in G_2_/M phase by treating the cell with nocodazole (100 ng/ml, Abcam) for 12 h [18]. After soft wash with cold PBS, the CD38^+/+^ or CD38^−/−^ CAMs was incubated with vehicle (Vehl) or baf for 24 h and then collected by trypsinization. Furthermore, the CAMs was harvested by centrifugation on 700 rpm at 4°C for 10 min and washed twice with PBS. The cells were then fixed in ice-cold 70% ethanol for 30 min at 4℃ and washed twice with PBS. The cells were treated with ribonuclease (100 μg/ml, Abcam) followed by addition of 200 μL propidium iodide (PI, 50 μg/ml, Abcam). Samples were analyzed using a Guava Easycyte Mini Flow Cytometry System and the Guava acquisition and analysis software (Guava Technologies, Hayward, CA). The cell number in G0/G1, S or G2/M phase was divided by the total cell number (G0/G1+S+G2/M) to indicate the percentage of cells in specific phases.

### RNA Interference of p62

The siRNAs targeting p62 (5′-AATTCTCCGAACGTGTCACGT-3′) or scrambled siRNA (Cell Signaling Technology, Denvers, MA) were transiently transfected using the siLentFect Lipid Reagent (Bio-Rad, Hercules, CA) according to the manufacturer’s instructions.

### Quantitative real-time PCR (qRT-PCR)

Total RNA from cells was extracted with TRIzol reagent (Invitrogen, Carlsbad, CA) according to the manufacturer’s protocol. One-microgram aliquots of total RNA from each sample were reverse-transcribed into cDNA by using a first-strand cDNA synthesis kit (Bio-Rad). Equal amounts of the reverse transcriptional products were subjected to PCR amplification on a Bio-Rad iCycler system (Bio-Rad). All the primers used in the study were synthesized by Operon (Huntsville, AL) and the sequences were as follows: CDK1 sense, 5′-TACGGCCGTCTACGTCTTCT-3′; CDK1 antisense, 5′-CGCAGATCACACTCCTCAAA-3′; β-actin sense 5′-TCGCTGCGCTGGTCGTC-3′; β-actin antisense 5′-GGCCTCGTCACCCACATAGGA-3′.

### Gene transfection in mouse coronary artery by ultrasound-microbubble technique

Plasmid DNA or shRNA (200 μg) was freshly prepared in 300 μL of saline with 20% microbubble (Optison; GE Healthcare, Chalfont St. Giles, UK). After anesthesia by 2% of isoflurane, the anterior chest was shaved and the left femoral vein was exposed as well. The plasmid mixture was directly injected into the femoral vein. Simultaneously, transthoracic ultrasound insonation (Sonitron 2000; Rich-Mar, Inola, OK) was performed through a 6-mm diameter probe with an input frequency of 1 MHz, an output intensity of 1.0 to 2.0 W/cm^2^ and a pulse duty ratio of 10% to 50% for a total of 180 seconds with 30-second intervals. After closing the wound, the mouse remained on the heating board until recovery. The mice were sacrificed 4 days later and the heart was harvested as described above.

### Immunofluorescence staining of coronary arteries

Immunofluorescence studies of mouse hearts was performed with frozen slides using corresponding immunofluorescence labeled antibodies as described previously [19]. Briefly, the tissues of hearts with coronary arteries were frozen in Tissue-Tek OCT, cut by cryostat into 10 μm sections and mounted on Superfrost/Plus slides for immunofluorescence staining. After fixation with acetone, the slides were incubated with the indicated antibodies (1:50 dilution) overnight at 4°C. The slides were then washed and labeled with corresponding Alexa-488 conjugated secondary antibodies (Invitrogen) used at a dilution of 1:200. Samples were stained for 60 min, washed, mounted and subjected to confocal microscopic analysis (Fluoview FV1000, Olympus, Japan).

### Statistics

Data are presented as means±SEM. Significant differences in mean values between- and within-multiple groups were examined using analysis of variance (ANOVA), and any significant difference revealed by this procedure were further investigated using Tukey’s multiple-range test. Student’s *t*-test was used to detect significant difference between two groups. The statistical analysis was performed by the Sigmastat 3.5 software (Systat Software, Chicago, IL, USA). P<0.05 was considered statistically significant.

## Results

### Autophagy defect promoted dedifferentiation of CAMs

**Fig. 1.**
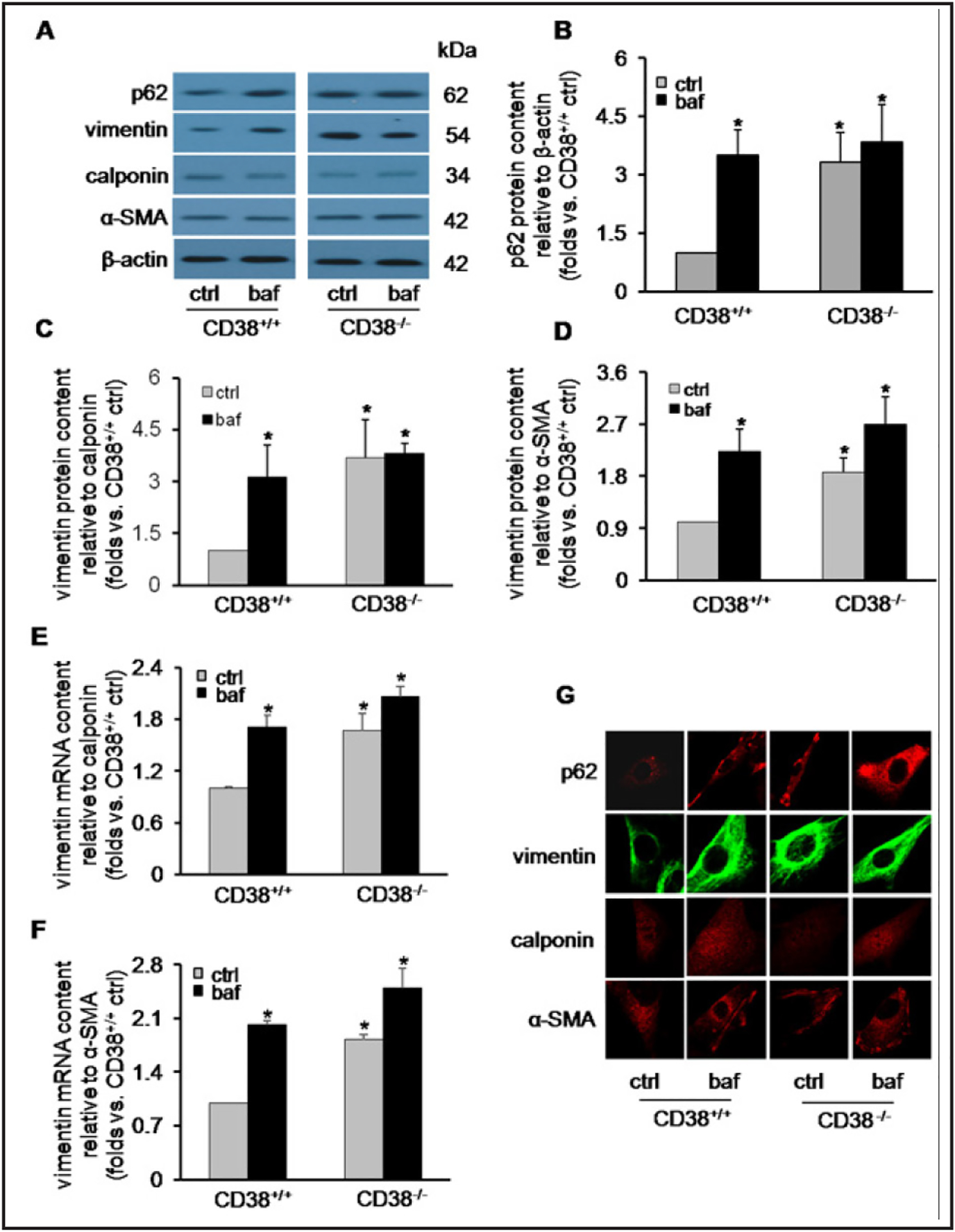
CD38 gene deficiency or bafilomycin (baf)-treatement induces dedifferentiation in primary cultured coronary arterial myocytes (CAMs) of mice. (A) Representative western blot gel documents, (B, C and D) summarized densitometric data showing p62 protein expression and relative protein abundance of vimentin to calponin or α-smooth muscle actin (SMA) in CD38^+/+^ or CD38^−/−^ CAMs under control (ctrl) or baf treatment. (E and F) Summarized data showing relative mRNA abundance of vimentin to calponin or α- SMA. (G) Representative confocal microscopic fluorescent images showing protein content of p62, vimentin, calponin and α-SMA. Data are shown as means±SEM, n=5, *P<0.05 *vs*. CD38^+/+^ ctrl.

To define the effect of autophagy disorder on the phenotype change, we examined the protein and mRNA expression of p62, vimentin, a marker of dedifferentiation, as well as α-smooth muscle actin (α-SMA) and calponin, two markers of contractile phenotype in CAMs. As shown in Fig. 1A and 1B, both CD38^−/−^ and bafilomycin (baf), an inhibitor of lysosomal function treated CAMs had a significantly higher level of p62 or vimentin protein expression than CD38^+/+^ CAMs under control condition. However, the protein expression of α-SMA or calponin did not changed obviously by CD38 gene deficiency or baf treatment, so the ratio of vimentin protein level to α-SMA or calponin was increased significantly in CD38^−/−^ and baf treated CAMs (Fig. 1C and 1D, P<0.05) as compared to CD38^+/+^ control CAMs. The ratio of vimentin mRNA level to α-SMA or calponin was also enhanced by CD38 gene deficiency or baf treatment (Fig. 1E and 1F, P<0.05). The immunofluorescent detection of p62, vimentin, α-SMA or calponin protein showed similar change as western blot examination (Fig. 1G).

### p62 gene silencing reversed the phenotype transition and proliferation of CD38^−/−^ or baf treated CAMs

**Fig. 2.**
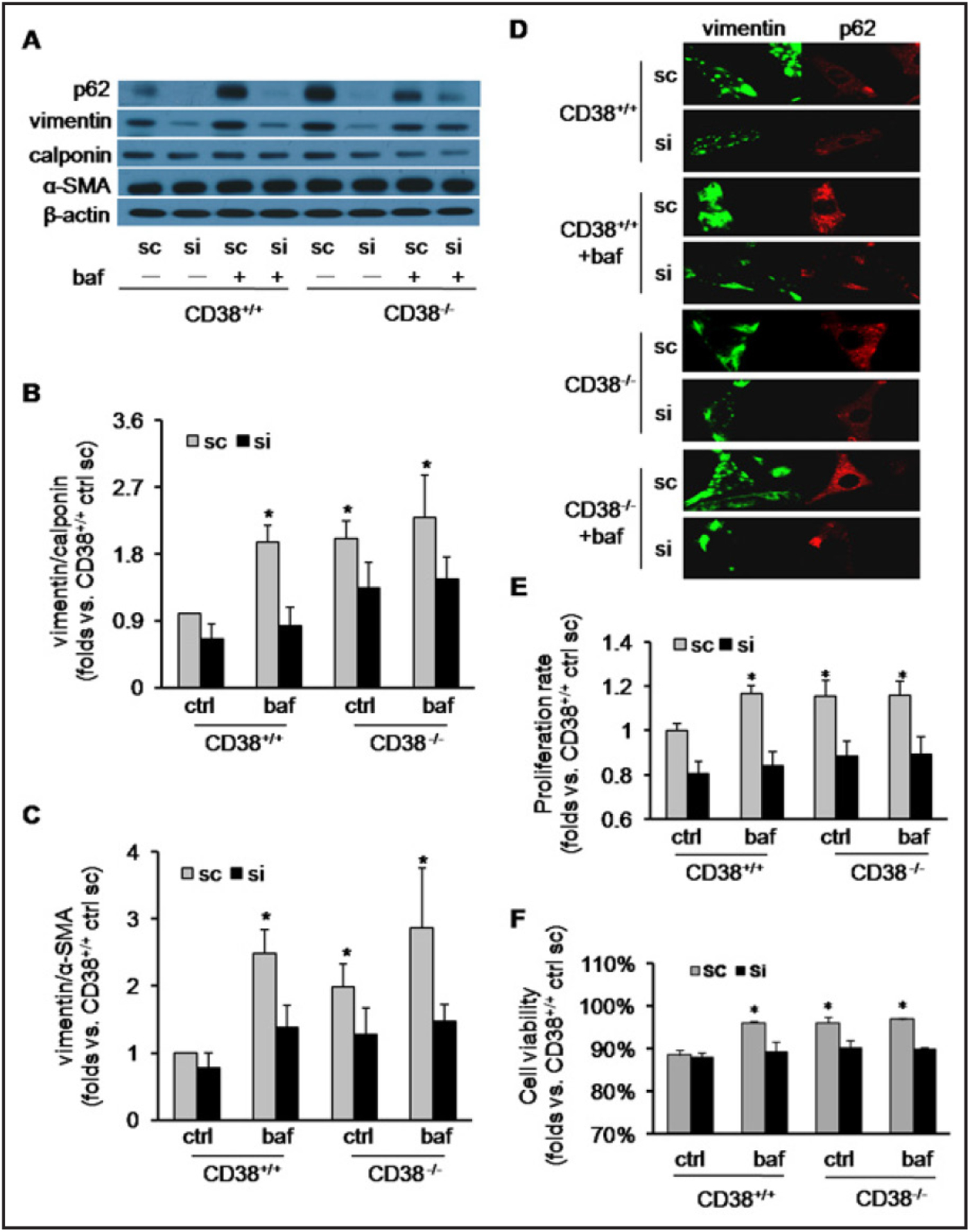
P62 gene silencing reverses phenotype transition and proliferation in CD38^−/−^ or baf treated CAMs. (A) Representative western blot gel documents, (B and C) summarized densitometric data and (D) representative confocal microscopic fluorescent images showing relative abundance of vimentin to calponin or α-SMA in CD38^+/+^ or CD38^−/−^ CAMs under ctrl or baf treatment transfected by scrambled sRNA (sc) or p62 siRNA (si). (E and F) Summarized data showing the relative proliferation rate of CD38^+/+^ or CD38^−/−^ CAMs detected by MTS (E) or Trypan blue (F) assay. Data are shown as means±SEM, n=5, *P<0.05 vs. CD38^+/+^ ctrl with sc transfection.

Since p62 accumulation is associated with proliferation, migration and poor differentiation of various cells [10], we examined the role of p62 in phenotype transition of CAMs with deranged autophagy. We found p62 siRNA transfection knocked down the p62 protein expression remarkably which reverted the increased vimentin expression in CD38^−/−^ or baf treated CAMs significantly, while the expression of calponin or α-SMA was not changed (Fig. 2A). So the ratio of vimentin to calponin or α-SMA was reversed after p62 expression was knocked down (Fig. 2B and 2C). Immunofluorescent studies showed similar result that both p62 and vimentin were upregulated in CD38 deficient or baf treated CAMs which was corrected by p62 gene silencing (Fig. 2D). Furthermore, proliferation rate of CAMs was increased following autophagy defect and was restored after p62 siRNA was transfected (Fig. 2E and 2F).

### p62 gene silencing reversed the cell cycle progression of CD38^−/−^ or baf treated CAMs

**Fig. 3.**
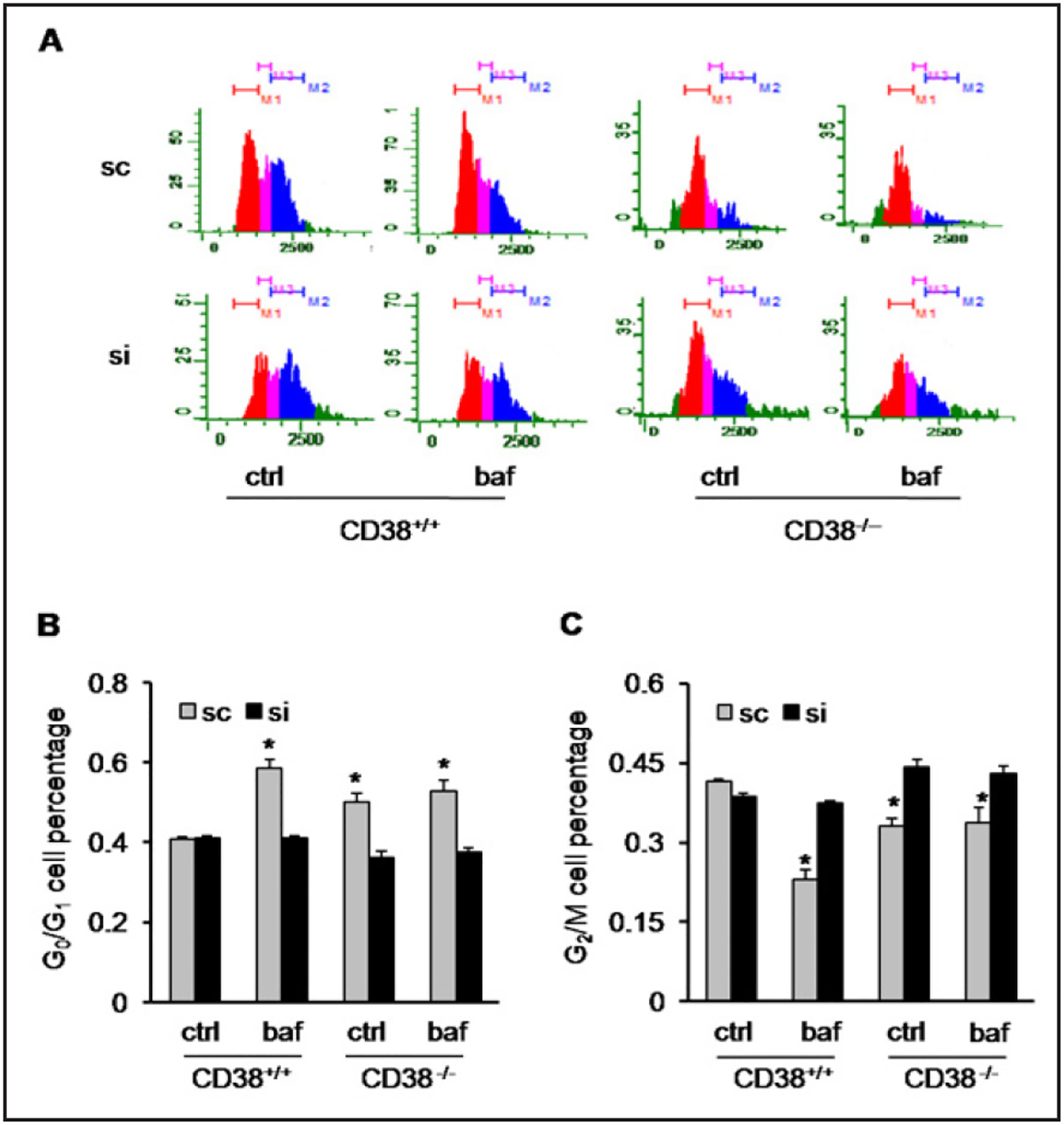
CD38 gene deficiency or baf treatment triggers cell cycle progress by through p62 accumulation in CAMs. (A) Representative flow cytometric image of cell cycle analyses of CD38^+/+^ and CD38^−/−^ CAMs under ctrl or baf treatment with sc or si transfection which indicated G_0_/G_1_ (red, M1), S (pink, M3) and G_2_/M (blue, M2) phases of cell cycle. (B and C) summarized data demonstrating the percentage of cell at G_0_/G_1_ or G_2_/M phase relative to total cell number. Data are shown as means ± SEM, n=4, *P<0.05 *vs*. CD38^+/+^ ctrl with sc transfection.

As cell cycle progress determines proliferation rate of cells, we then investigated cell cycle changes in CAMs upon CD38 gene deficiency or lysosomal dysfunction. The original flow cytometric image of cell cycle analysis in CAMs showed three distinct signals representing G_0_/G_1_ (red, M1), S (pink, M2) and G_2_/M (blue, M3) phases (Fig. 3A). As compared to untreated CD38^+/+^ cell, the percentage of cell at G_0_/G_1_ phase was increased significantly in CD38^−/−^ or baf treated CAMs (*P*<0.05, Fig. 3B), while the fraction of cell at G_2_/M phase was significantly decreased (*P*<0.05, Fig. 3C), both of which could be normalized by p62 siRNA transfection.

### p62 gene silencing reversed the changes of CDK1 ubiquitinylation and degradation in CD38^−/−^ or baf treated CAMs

**Fig. 4.**
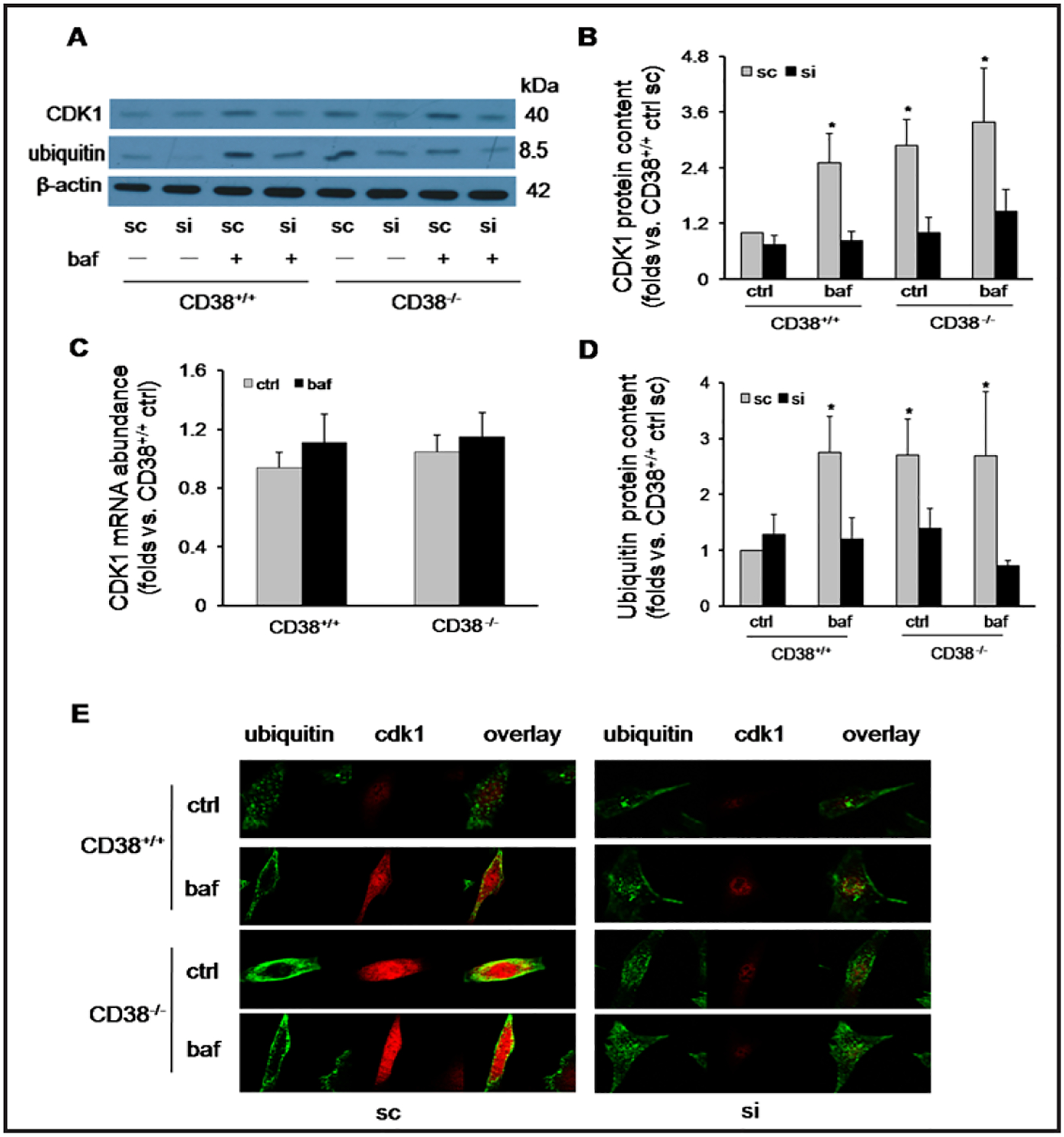
P62 gene silencing restores the ubiquitinylation and degradation of cyclin-dependent kinase 1 (CDK1) compromised in CD38^−/−^ or baf treated CAMs. (A) Representative western blot gel documents, (B and D) summarized densitometric data showing protein abundance of CDK1 or free ubiquitin in CD38^+/+^ or CD38^−/−^ CAMs under ctrl or baf treatment transfected by sc or si. (C) Summarized data showing mRNA abundance of CDK1 in CD38^+/+^ or CD38^−/−^ CAMs under ctrl or baf treatment. (E) Representative confocal microscopic fluorescent images showing protein expression and distribution of CDK1 and free ubiquitin in CD38^+/+^ or CD38^−/−^ CAMs under ctrl or baf treatment with sc or si transfection. Data are shown as means ± SEM, n=5, *P<0.05 vs. CD38^+/+^ ctrl with sc transfection.

The proper control of CDK1 is crucial during the mitotic prophase, metaphase and anaphase of cell [20]. Given the alteration of proliferation and cell cycle in CD38^−/−^ or baf treated CAMs, we next measured the content of CDK1 in CAMs. CDK1 protein level was significantly upregulated (*P*<0.05) by CD38 deficiency or baf treatment. These alterations were normalized by p62 gene silencing (Fig. 4A and 4B). However, neither CD38 deficiency nor baf treatment changed the mRNA levels of CDK1 (Fig. 4C), indicating a posttranslational control of CDK1 expression by autophagy defect. As CDK1 is degraded mainly through the ubiquitin-proteasome pathway [21], we then tested whether ubiquitinylation of CDK1 was affected in CD38^−/−^ or baf treated CAMs. We first detected the protein level of free ubiquitin and found a significant increase of it in cells lacking CD38 or treated with baf, which was reversed by p62 gene silencing (Fig. 4D). We also performed immunofluorescent experiments to investigate distribution of CDK1 and ubiquitin in the cells. As shown in Fig. 4E, in untreated CD38^+/+^ CAMs, the CDK1 was mainly localized in the nucleus and cytosol showing co-localization with ubiquitin. However, in CD38^−/−^ or baf treated CAMs, ubiquitin was translocated to the cell membrane prominently, while the co-localization of CDK1 and ubiquitin was greatly reduced, which was accompanied by the higher CDK1 protein level in these cells. Transfection of CAMs with p62 siRNA normalized the cellular distribution of ubiquitin in CD38 deficient or baf treated cells from cell membrane to nucleus and cytosol. In the mean time, the co-localization of ubiquitin with CDK1 and the protein levels of CDK1 were recovered.

### CD38 gene deficiency or chloroquine (chlo) treatment induced phenotype transition and proliferation in coronary artery of mice

**Fig. 5.**
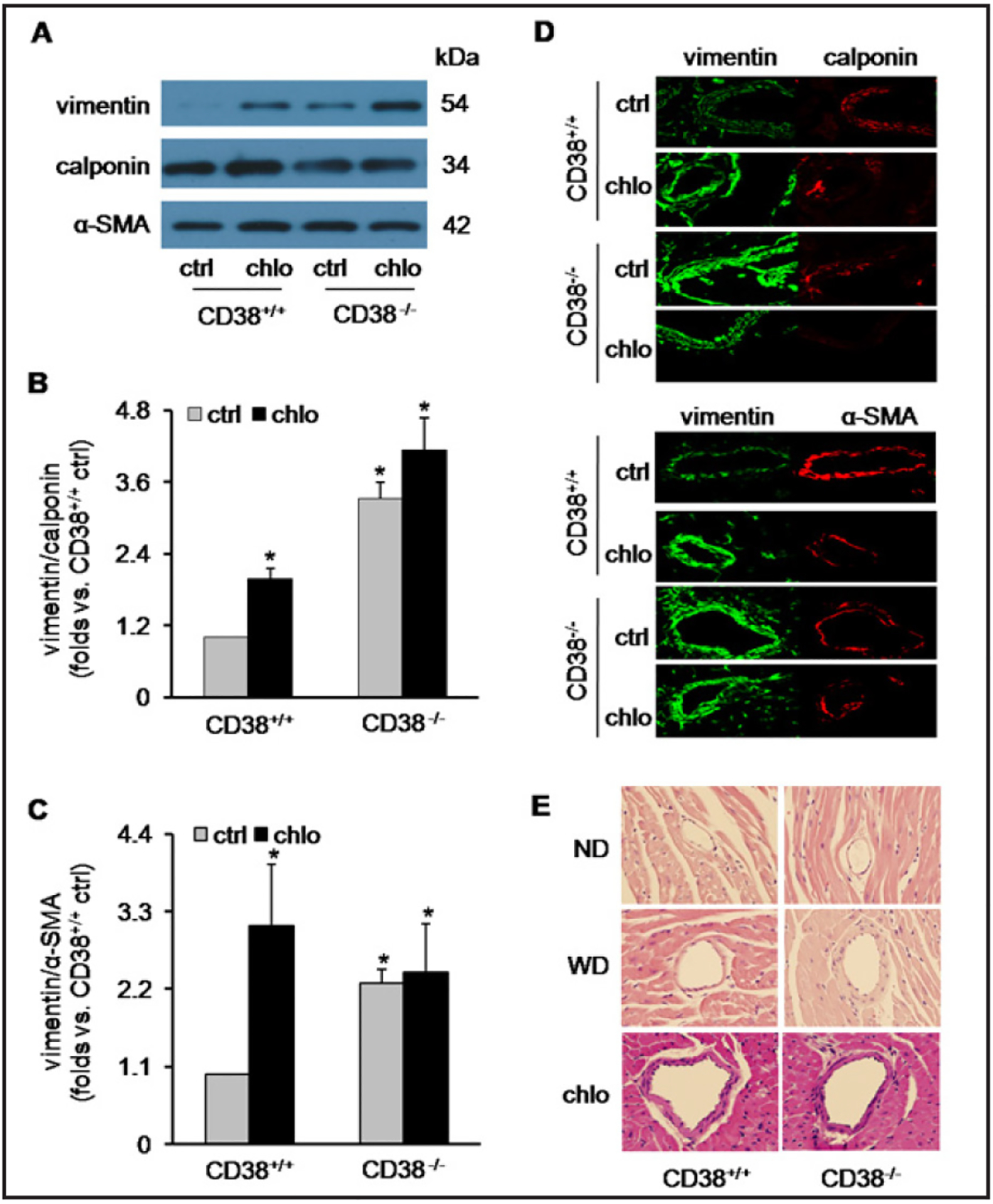
CD38 gene deficiency or chloroquine (chlo) treatment provokes dedifferentiation phenotype switching and proliferation in coronary arteries of mice. (A) Representative western blot gel documents, (B and C) summarized densitometric data and (D) representative confocal microscopic fluorescent images showing relative abundance of vimentin to calponin or α-SMA in coronary arteries of CD38^+/+^ or CD38^−/−^ mice under ctrl or chlo treatment. (E) Representative image of hematoxylin-eosin staining of paraffin sections of heart indicating the wall thickness of coronary arteries in CD38^+/+^ and CD38^/−^ mice fed normal diet (ND) or a high-fat Western diet (WD) for 10 weeks or upon chlo treatment. n=5, *P<0.05 vs. CD38^+/+^ ctrl.

We further conducted *in vivo* tests to examine the phenotype change of CAMs due to altered autophagic flux. As shown in Fig. 5A, both CD38^−/−^ gene deficiency and intraperitoneal injection of chol, a lysosomal functional inhibitor, increased protein level of vimentin. However, the protein expression of calponin or α-SMA in the coronary arterial wall did not change (Fig. 5A). So the ratio of vimentin to calponin or α-SMA was substantially increased in mice lacking CD38 gene or receiving chol treatment (*P*<0.05, Fig. 5B and 5C). Immunofluorescent studies confirmed the increased expression of vimentin as well as the decreased expression of calponin or α-SMA in arterial wall of CD38^−/−^ or chlo treated mice (Fig. 5D). In addition, the coronary arterial wall was much thicker in CD38^−/−^, Western diet (WD) fed or chol treated mice than in CD38^+/+^ or normal diet (ND) fed mice (Fig. 5E).

### CD38^−/−^ or chlo treated mice showed higher expression of CDK1 and ubiquitin in coronary artery

**Fig. 6.**
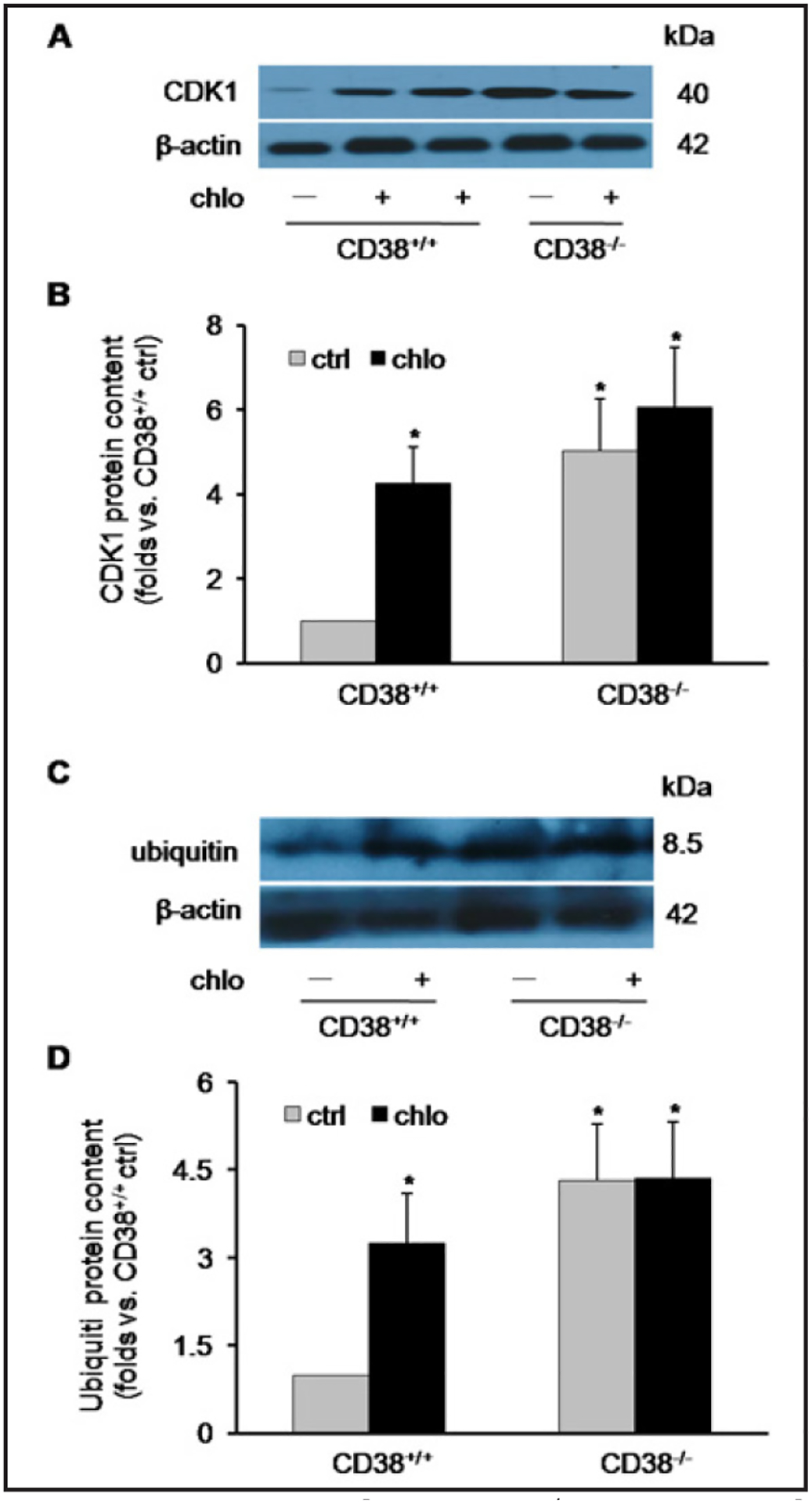
CD38 gene deficiency or chlo treatment prevents ubiquitinylation and degradation of CDK1 in coronary arteries of mice. (A and C) Representative western blot gel documents, (B and D) summarized densitometric data showing protein abundance of CDK1 or free ubiquitin in coronary arteries of CD38^+/+^ or CD38^−/−^ mice under ctrl or chlo treatment. n=4, *P<0.05 vs. CD38^+/+^ ctrl.

Next, we investigated the change of CDK1 ubiquitinylation in the coronary arterial wall of mice. As shown in the result of western blot analysis, the coronary artery of CD38^−/−^ or chol-treated mice had more CDK1 protein expression than CD38^+/+^ or untreated mice (*P*<0.05, Fig. 6A and 6B). In the meantime, the free ubiquitin protein level was increased significantly by CD38 gene deficiency or chol treatment (*P*<0.05, Fig. 6C and 6D).

### p62 gene silencing reversed phenotype switching and CDK1 ubiquitinylation in coronary artery of CD38^−/−^ or chlo treated mice

**Fig. 7.**
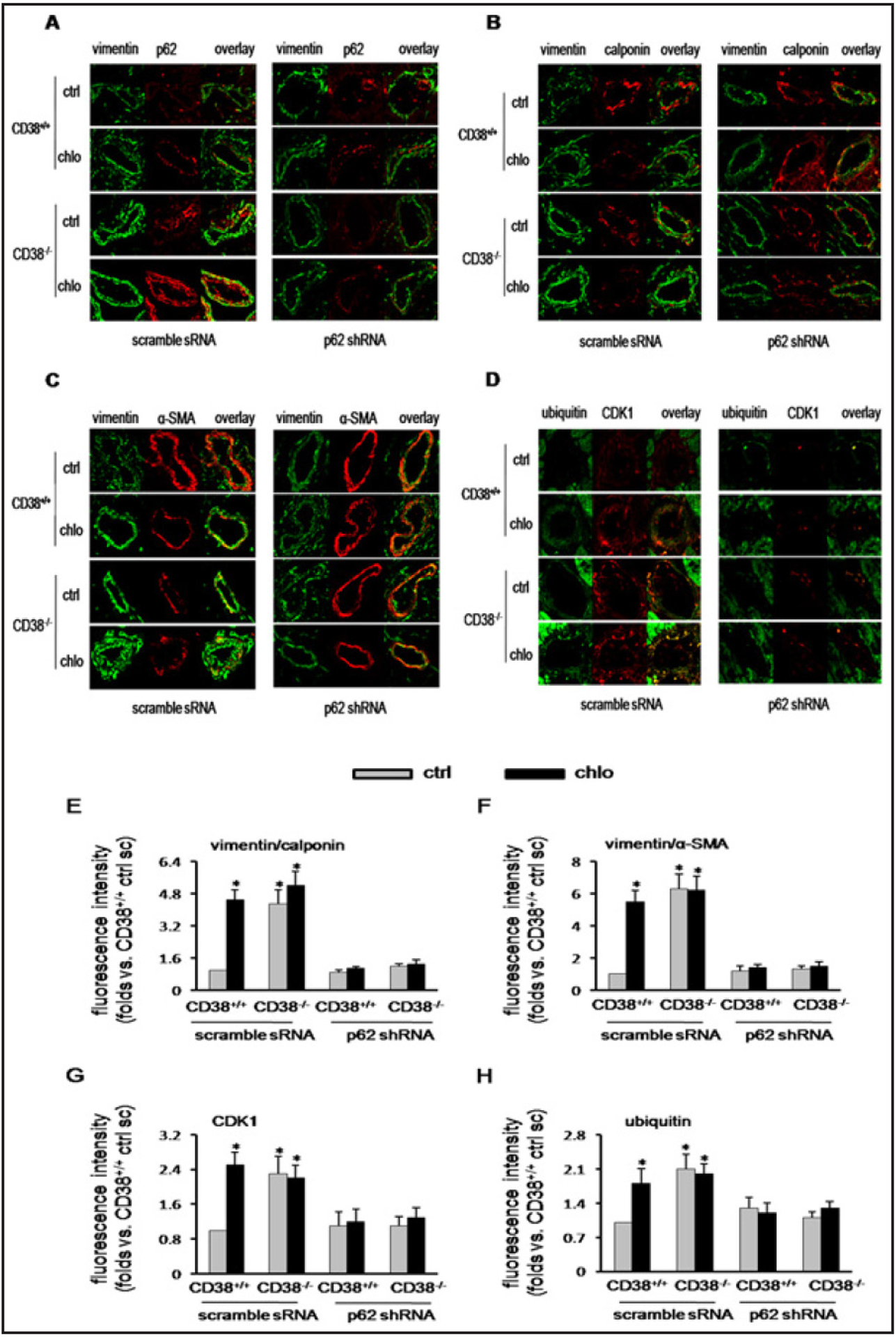
P62 gene silencing corrects the phenotype switching and compromised CDK1 ubiquitinylation and degradation in coronary arteries of CD38^−/−^ or chlo-treated mice. (A) Representative confocal microscopic fluorescent images showing efficiency of p62 shRNA *in vivo* transfection. (B, C, E and F) Representative confocal microscopic fluorescent images and summarized data showing relative abundance of vimentin to calponin or α-SMA in coronary arteries of CD38^−/−^ or chlo-treated mice transfected with scramble sRNA (sc) or p62 shRNA. (D, G and H) Representative confocal microscopic fluorescent images and summarized data indicating protein abundance of CDK1 or free ubiquitin in coronary arteries of CD38^+/+^ or CD38^−/−^ mice under ctrl or chlo treatment with scramble sRNA or p62 shRNA transfection. n=4, *P<0.05 vs. CD38^+/+^ ctrl.

Next, we investigated the role of p62 in phenotype change and CDK1 ubiquitinylation of the coronary arterial wall of mice lacking CD38 or after chol-treatment. We used p62 shRNA *in vivo* transfection to knock down the expression of p62 in coronary arterial wall of mice which showed high efficiency (Fig. 7A). As shown in the original and summarized data of confocal immunofluorescent examination, the ratio of vimentin to calponin (*P*<0.05, Fig. 7B and 7E) or α-SMA (*P*<0.05, Fig. 7C, and 7F) upregulated in CD38^−/−^ or chlo-treated mice was normalized by p62 shRNA transfection. Meanwhile, the increased level of CDK1or ubiquitin (*P*<0.05, Fig. 7D, 7G and 7H) in coronary arteries of CD38^−/−^ or chlo-treated mice was also reversed after p62 gene was silenced.

## Discussion

Autophagy defect promotes proliferation and phenotype transition of VSMCs which is closely related to the occurring of atherosclerosis and the mechanism is not fully understood. CD38 deficiency or lysosomal dysfunction blocks autophagy by attenuating autophagosome trafficking and fusion with lysosomes. The present study demonstrated that p62 was accumulated in CD38^−/−^ CAMs or upon lysosomal dysfunction which contributed to proliferation and dedifferentiation of the cells. p62 might exert functions through diminishing ubiquitinylation and degradation of CDK1 to promote G_2_/M phase progression during cell cycle.

VSMCs are located in the media layer of arteries which expresses contractile proteins to regulate vascular tone, normal status of which is crucial for the proper functioning of arteries. However, in pathological conditions such as hypertension, restenosis, and atherosclerosis, VSMCs may transit from a contractile to a dedifferentiated or synthetic phenotype, which elicits their proliferation and migration into the intima and induces synthesis of extracellular matrix proteins [22, 23]. Phenotypic transition of VSMCs has been considered to be a key initiator of atherogenesis and the happening of it relates to multiple mechanisms among which the autophagy defect has been testified in many studies [24, 25]. However, how the defective autophagy evokes proliferation and phenotype switching of VSMCs is largely unknown.

CD38 is a multifunctional enzyme responsible for the production and metabolism of cADPR and NAADP in VSMCs. The progress of autophagy depends a lot on the CD38 triggered Ca^2+^ release from lysosomal stores and the following Ca^2+^-induced Ca^2+^ release through inositol 1,4,5-trisphosphate receptors and ryanodine receptors on the sarcoplasmic reticulum [26, 27]. In our former studies, we demonstrated that in CAMs or coroanry arterial wall of mice lacking CD38 gene, autophagic flux was markedly impaired which is mainly due to the suppression of autophagosome trafficking and fusion with lysosomes [6, 28, 29].

In the present study, we first demonstrated that a defective autophagy in CAMs following lysosomal dysfunction or CD38 gene deficiency induced phenotypic transition from contractile to synthetic status which contributed to atherogenesis in WD-fed mice. The results indicate that the normal lysosomal function and autophagy are important for maintenance of contractile state of VSMCs in coronary artery which is consistent with the viewpoint on the role of autophagy in vascular atherosclerotic injury. It is well accepted that autophagy is a cell survival mechanism in almost all mammalian cells promoting degradation of long-lived proteins and excessive or dysfunctional organelles. Under physiological conditions, it works in a nonstop, reparative and life-sustaining way to maintain normal cellular homeostasis [2]. Autophagy may have both protective and detrimental roles during atherosclerosis, depending upon the status of autophagy or stages of atherosclerosis [2, 30]. In addition, it in different cells may play different roles in atherosclerosis. For example, increased autophagic death in macrophages attenuates the foam cell formation, reducing atherosclerotic injury [30]. However, excessive activation of autophagy in endothelial cells (ECs) may lead to damage of the endothelium enhancing atherogenic injury [30]. In VSMCs, enhanced autophagy may induce differentiated, quiescent, and contractile phenotype transition decreasing cell proliferation and preventing fibrosis [2, 6, 30]. Nevertheless, excessive autophagy in VSMCs results in cell death increasing the instability of atherosclerotic plaques [30].

To explore the mechanisms responsible for the phenotype transition of CAMs following defective autophagy, we worked on the signaling pathway centered on p62, a scaffolding adaptor protein, given it is selectively degraded by autophagy and could be accumulated in VSMCs when autophagy progress is blocked [4]. The p62 contains several protein-protein interacting domains that are key motifs for selective autophagy including a N-terminal PB1 domain, a zinc finger (ZZ) domain, a LIR motif, a TRAF6-binding domain (TB) and a C-terminal UBA domain. PB1 is in charge of self-oligomeration or hetero-oligomeration with other proteins. Through LIR motif and UBA domain, p62 interacts with ATG8 family and ubiquitinated proteins which is essential for the formation of autophagosomes and degradation of proteins [8]. However, excessive p62 is harmful as shown by the findings that p62 accumulation was associated with neurodegeneration [12, 13], cancer cell proliferation and migration [11] or podocytes epithelial-to-mesenchymal transition [31]. Similarly, in the present work, by using genetic and pharmacological manipulations, we found that p62 accumulation induced by CD38 gene deficiency or lysosomal dysfunction was a major contributor to the proliferation and dedifferentiation change of CAMs.

Since p62 plays an important role in cell cycle regulation which could lead to the cellular transformation [18], we supposed p62 accumulation after autophagic derangement may induce phenotypic transition of CAMs though its modulation on cell cycle. In the present study, we found that CD38 gene deficiency or lysosomal disruption decreased the percentage of CAMs in G_2_/M phase and at the same time increased that in G_0_/G_1_ phase, indicating the G_2_/M progress has been boosted. This cell cycle changes may underlie the proliferation and phenotype switching of CAMs. Furthermore, we found p62 accumulation may trigger this cell cycle alteration for as compared to scramble sRNA, transfection of p62 siRNA to CD38^−/−^ or baf treated CAMs markedly increased and decreased the cell number in G_2_/M and G_0_/G_1_ phase, respectively. These results were in line with the effect of p62 gene silencing on proliferation and phenotype we got afore. In this regard, several independent studies have proved the critical role of cell cycle change in determining higher proliferation and phenotype switching of VSMCs [32]. In pulmonary arterial smooth muscle cells, hypoxia-induced proliferation was associated with G_2_/M cell cycle progression via phosphatidylinositol 3-kinase/Akt pathway [33]. Leptin induced proliferation of VSMCs via promoting transition of cell cycle from G_1_ to S phase [34]. Treating human VSMCs with meclofenamic acid in combination with pharmacologic cyclooxygenase-2 inhibition reversed their proliferation, colony formation, and migration, which was related to increased G_2_/M phase share [35].

CDK1, by binding with cyclin B1, acts as key regulator of cell entering and progressing through cell cycle [20, 36, 37]. Cyclin B1 reaches the highest level in the S phase and is degraded at the end of mitosis after which the CDK1 is normally inactivated [38, 39]. We then tested whether the defective autophagy altered CDK1 expression resulting in the phenotypic transition of CAMs. It was found that the CDK1 protein content was significantly increased in CD38 deficient CAMs or upon lysosomal dysfunction, concurring with the alterations in cell cycle, proliferation and phenotype transition of CAMs. Also, the changes in CDK1 were reversed by p62 siRNA silencing, indicating p62 was critical in modulation of CDK1in the condition. Since we did not detect any change in CDK1 mRNA quantity, it might be the posttranslational regulation or diminished degradation of CDK1 existing with autophagic flux blocking.

We further addressed whether the changes in CDK1 after autophagy defect was related to the ubiquitinylation-mediated protein clearance in CAMs. Ubiquitin is a small regulatory protein with a molecular weight of 8.5 kDa present in almost all tissues of eukaryotic organisms. Ubiquitinylation is the initial step for degradation of multiple proteins via either autophagy or proteasome pathway [40]. During ubiquitinylation, ubiquitin would be bound to the target protein which might increase the molecular weight of it and the content of free ubiquitin would be reduced [41, 42]. So in the study, we tried to analyze the level of ubiquitinylation of CDK1 by measuring both abundance of free ubiquitin as well as the relative distribution of ubiquitin or CDK1 in CAMs. We found the autophagy defect enhanced the protein level of free ubiquitin, and at the same time, drove it from cytosol and nucleus to cell membrane deviating from where the CDK1 was located. Our results indicate that the ubiquitinylation of CDK1 has been prevented in CD38^−/−^ or baf treated CAMs. Furthermore, we found the change of both protein level and distribution of ubiquitin could be reversed by p62 gene silencing which suggested p62 accumulation due to autophagy defect prevents the conjugation of ubiquitin to CDK1, blocking its degradation by the cellular proteasome. The finding is consistent with the previous reports that the excessive p62 due to autophagy defect inhibits the clearance of ubiquitinated proteins destined for proteasomal degradation by delaying their delivery to proteases [40, 43].

In summary, the present study demonstrated that the autophagy impairment upon CD38 deficiency or lysosomal dysfunction resulted in p62 accumulation, which activated dedifferentiated phenotype transition and proliferation of CAMs. This p62-mediated effect was attributable to compromised ubiquitinylation and degradation of CDK1 and enhanced G_2_/M cell phase progress during cell cycle. Our results might define a novel mechanism linking autophagy defect and phenotype transition of VSMCs in response to pathogenic stimulations.
